# Supplementary material for: Association between oral microbiome and seven types of cancers in East Asian population: a two-sample Mendelian randomization analysis
Source: Front Mol Biosci. 2023 Nov 21;10:1327893. doi: 10.3389/fmolb.2023.1327893 (PMC10702768; doi:10.3389/fmolb.2023.1327893)
Supplement: Supplementary file 1 [file DataSheet1.ZIP › Supplementary Materials/MR plots for tongue/tongue═╝/Ovarian cancer/pheno.173_to_ovarian cancer_forest.pdf]

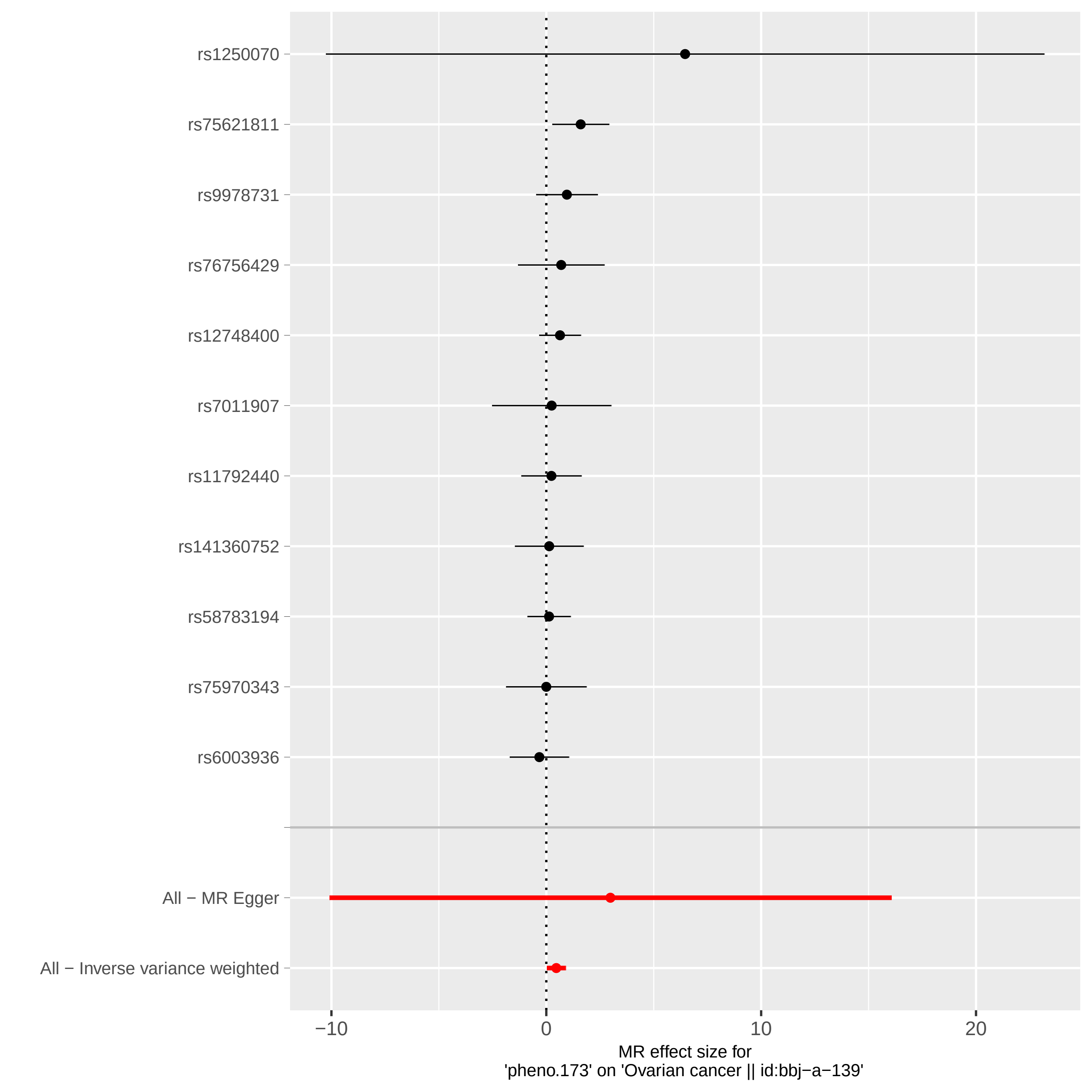
rs1250070
rs75621811
rs9978731
rs76756429
rs12748400
rs7011907
rs11792440
rs141360752
rs58783194
rs75970343
rs6003936
All − MR Egger
All − Inverse variance weighted
−10
0
10
20
MR effect size for
'pheno.173' on 'Ovarian cancer || id:bbj−a−139'
